# Case Report: Complete Necrosis of a Large Adrenocortical Cancer and Liver Metastases Achieved by Selective Arterial Embolization: A Case Study and Review of Literature

**DOI:** 10.3389/fendo.2021.677187

**Published:** 2021-04-30

**Authors:** Gergely Huszty, Attila Doros, Katalin Farkas, László Kóbori, Péter Reismann, Judit Tőke, Miklós Tóth, Peter Igaz

**Affiliations:** ^1^ Department of Transplantation and Surgery, Faculty of Medicine, Semmelweis University, Budapest, Hungary; ^2^ Department of Endocrinology and Department of Internal Medicine and Oncology, Faculty of Medicine, Semmelweis University, Budapest, Hungary; ^3^ Department of Internal Medicine and Oncology, European Reference Network for Rare Endocrine Diseases Health Care Provider (ENDO-ERN HCP), Faculty of Medicine, Semmelweis University, Budapest, Hungary; ^4^ MTA-SE Molecular Medicine Research Group, Eotvos Lorand Research Network, Budapest, Hungary

**Keywords:** adrenocortical cancer, liver metastasis, trans-arterial, embolization, necrosis

## Abstract

There is very limited experience regarding the interventional radiological treatment of adrenocortical cancer (ACC). We present the case of a 57-year-old female patient with a large, potentially unresectable left-sided ACC and two hepatic metastases. Both liver tumors were effectively treated by trans-arterial embolization (TAE), followed by TAE of the bulky primary tumor as a life-saving intervention necessitated by severe intratumoral bleeding. Surgical removal of the primary tumor revealed complete necrosis. The patient is considered tumor free after 3.5 years. To the best of our knowledge, this is the first report to show that even a primary ACC may be completely ablated by selective embolization, and the fourth to prove the curative potential of liver TAE for ACC metastases. This case highlights the potential of selective embolization in ACC treatment.

## Introduction

Adrenocortical cancer (ACC) is a rare malignancy with mostly bad prognosis. Most cases are diagnosed late, in the presence of distant metastases (1–8). The medical treatment options for ACC are rather limited, as mitotane is the only available drug specific for the adrenal cortex and its use associated with severe side effects and a narrow therapeutic range (1). Systemic combination chemotherapy with EDP protocol (etoposide-doxorubicine-cisplatin) improves survival (2), but the only hope for cure is still the effective (R0) removal of the tumor and its metastases (3–6). According to the current clinical practice, the removal of the primary tumor is indicated whenever possible together with removable oligo-metastases, if R0 seems to be feasible; moreover, excessive hormonal symptoms might justify a palliative resection in exceptional cases (1–7).

The use of ablative methods other than surgery for ACC are poorly documented in the literature. Here we present a case, where intraarterial embolization was effectively used both for liver metastases and a bulky primary tumor leading to complete necrosis and effective surgical removal. To the best of our knowledge, this is the first case to show that an ACC primary tumor may be completely ablated by selective embolization, together with liver metastases.

## Case Presentation

**Figure 1:**
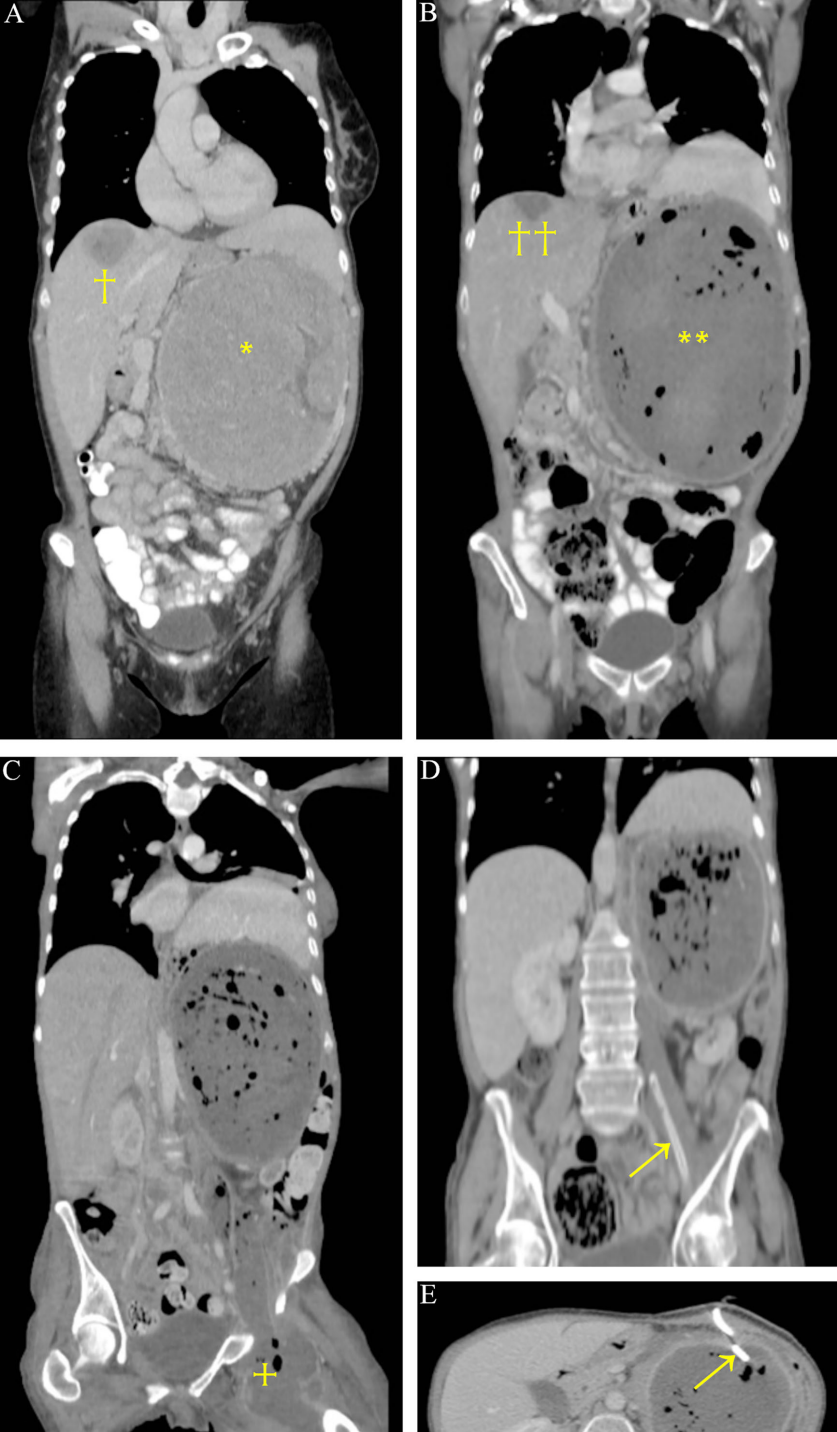
CT scan of 20 cm large left adrenocortical carcinoma (*) with segment 8 liver metastasis (†). The segment 7 tumor is not shown on this reconstruction **(A)**. Necrosis on CT after liver TAE (††) and embolization of the previously bleeding primary tumor (**) **(B)**. Abscess from the necrotic tumor reaching the thigh (+) **(C)**. Drains in the abscess (arrows); the descending part is already resolved **(D, E).**

A 57-year-old female patient presented with abdominal discomfort and 20 kilograms of weight loss without other specific complaints (body mass index (BMI): 19 kg/m^2^, serum albumin 35.5 g/L (normal 35-50)). On physical examination, a large palpable left upper quadrant non-tender mass was found. The patient was afebrile and had normal cardiopulmonary parameters. There were no preexisting chronic diseases in the medical history, nor were there any potential inherited genetic disorders in the family. The patient did not take any medications. Abdominal contrast-enhanced computed tomography (CT) showed a 20 cm subdiaphragmatic retroperitoneal tumor together with two metastases in segment 7 (29x21 mm) and segment 8 (52x43 mm) of the liver (**Figure 1A**). No lung or bone metastases were revealed.

**Figure 2:**
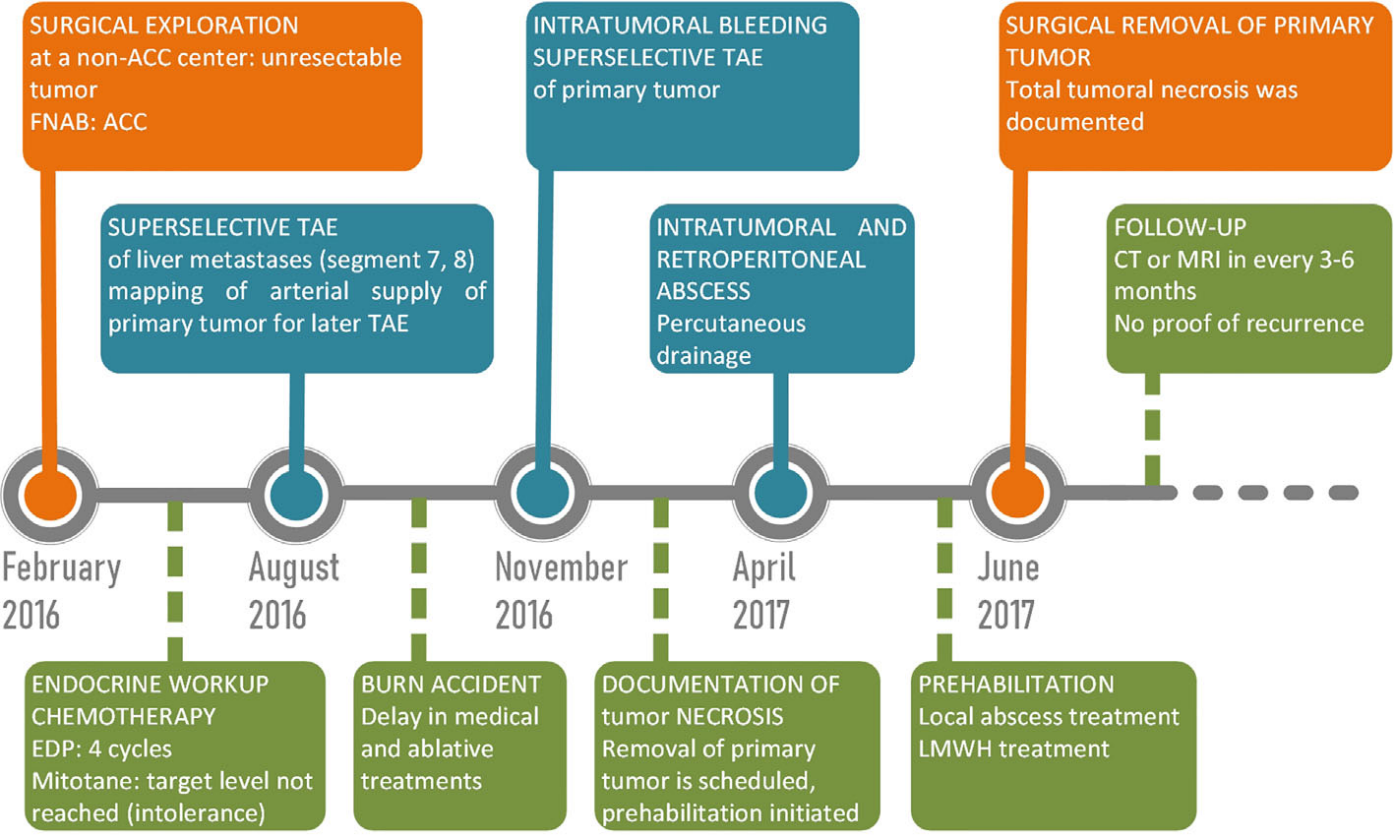
Timeline of case.

The timeline of the case with the most important therapeutic and diagnostic procedures is shown in **Figure 2**. The patient was surgically explored in a non-ACC center in February 2016. A large, strongly vascularized mass was found in the left subdiaphragmatic region; the tumor was judged unresectable, and therefore a fine needle aspiration biopsy (FNAB) sample was taken. Pathology showed a vimentin+, melan-A+, PanCK +, S100 –, DOG1 - adrenocortical cancer (Ki67-index was not defined). Her clinical stage corresponded to ENSAT (European Network for the Study of Adrenal Tumors) stage IV. There were no clinical signs of hormone overproduction. Detailed hormonal investigations showed a mild elevation in 17-OH-progesterone (310 ng/dL, normal range: 40-250) and dehydroepiandrosterone sulfate (DHEAS, 385.9 µg/dL, normal range: 130-330), whereas all cortisol-related laboratory parameters were in the normal range.

**Figure 3:**
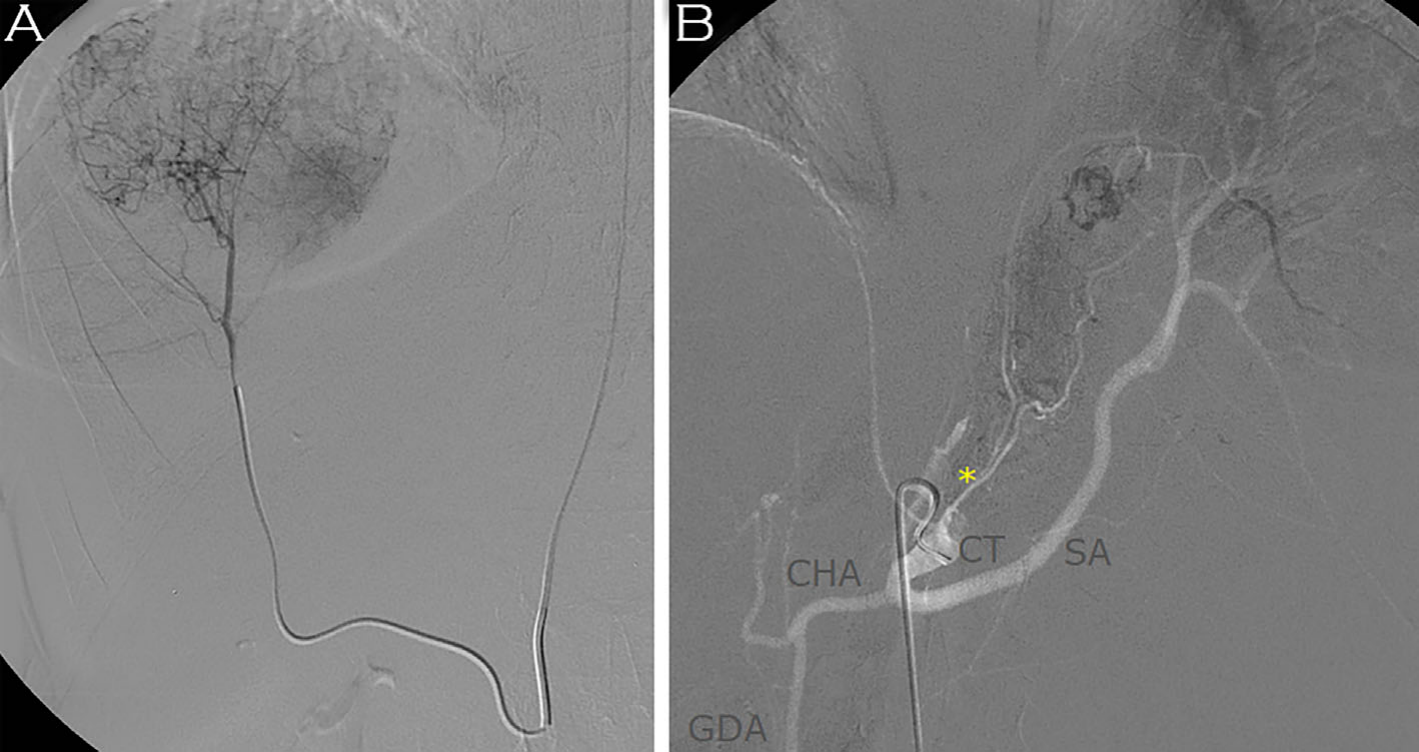
Embolization of the S8 tumor through the right replaced hepatic artery was achieved with 0.1mm PVA particles **(A)**. Embolization of the primary tumor through the left inferior phrenic artery was performed by 0.2mm PVA particles **(B)**. CT, celiac trunk; SA, splenic artery; CHA, common hepatic artery; GDA, gastroduodenal artery; *, dominant suprarenal artery from left inferior phrenic artery.

Adjuvant mitotane was initiated, but target level could not be reached because of serious, mostly gastrointestinal intolerance symptoms including nausea, severe malaise and diarrhea. EDP chemotherapy was initiated. The patient was treated with therapeutic LMWH (low molecular weight heparin) because of CT-documented portal vein thrombosis. After 4 cycles of EDP, staging CT in August 2016 showed significant growth in the S8 liver metastasis (d=7 cm) with unchanged primary. To affect tumor growth, palliative superselective trans-arterial embolization of both liver metastases was performed with 2 mL 0.1mm PVA particles through the replaced right hepatic artery arising from the superior mesenteric artery (**Figure 3A**). The dominant supplying artery of the primary tumor was documented, and a second intervention was scheduled. There were no adverse reactions following the TAE. However, an unfortunate home accident (6% second-degree burn) led to an unplanned delay in both medical and interventional radiological treatments. Soon after recovering from the skin burn, the patient was transferred to our center in November 2016 with a half-day history of diffuse abdominal tenderness, back pain, nausea and dizziness; she was seriously anemic (Hb 25 g/L, normal 120-150)) and showed obvious clinical signs of hemorrhagic shock. Together with life-saving fluid and blood resuscitation, CT was performed that showed intratumoral bleeding in the primary ACC and non-viable liver tumors. To control bleeding, hyperacute trans-arterial embolization of the primary tumor was performed with 1 mL 0.2mm PVA particles through the previously mapped dominant left suprarenal artery arising from the left inferior phrenic artery (**Figure 3B**). All the visceral, renal, and lumbar arteries were cannulated but did not seem to provide visible arterial supply to the tumor. The bleeding was stopped. One week later, the patient developed bronchopneumonia in the lower lobe of the left lung that was effectively treated with parenteral antibiotics (ceftriaxone), and the patient was stabilized, but her general condition was considered poor. She remained hypoalbuminemic after the burns, and her weight continuously dropped even after discontinuation of mitotane. The patient was considered to suffer from protein-energy-malnutrition (PEM) (Malnutrition Universal Screening Tool (9) score 4: high risk, BMI: 17 kg/m^2^, albumin 26.5 g/L). By this time, the otherwise non-tender abdominal mass that was still present on physical examination started to obviously cause difficulties in eating normal amounts of solid diet due to local gastric compression. The patient had severe concerns about a potential second bleeding but did not feel ready for a second operative exploration, which was mentioned to and discussed with her after the embolization. High caloric fluid supplementation diet was administered and prescribed before discharging her with stabile vital parameters. She was again referred to the multidisciplinary board of the university after a control CT in February 2017.

**Figure 4:**
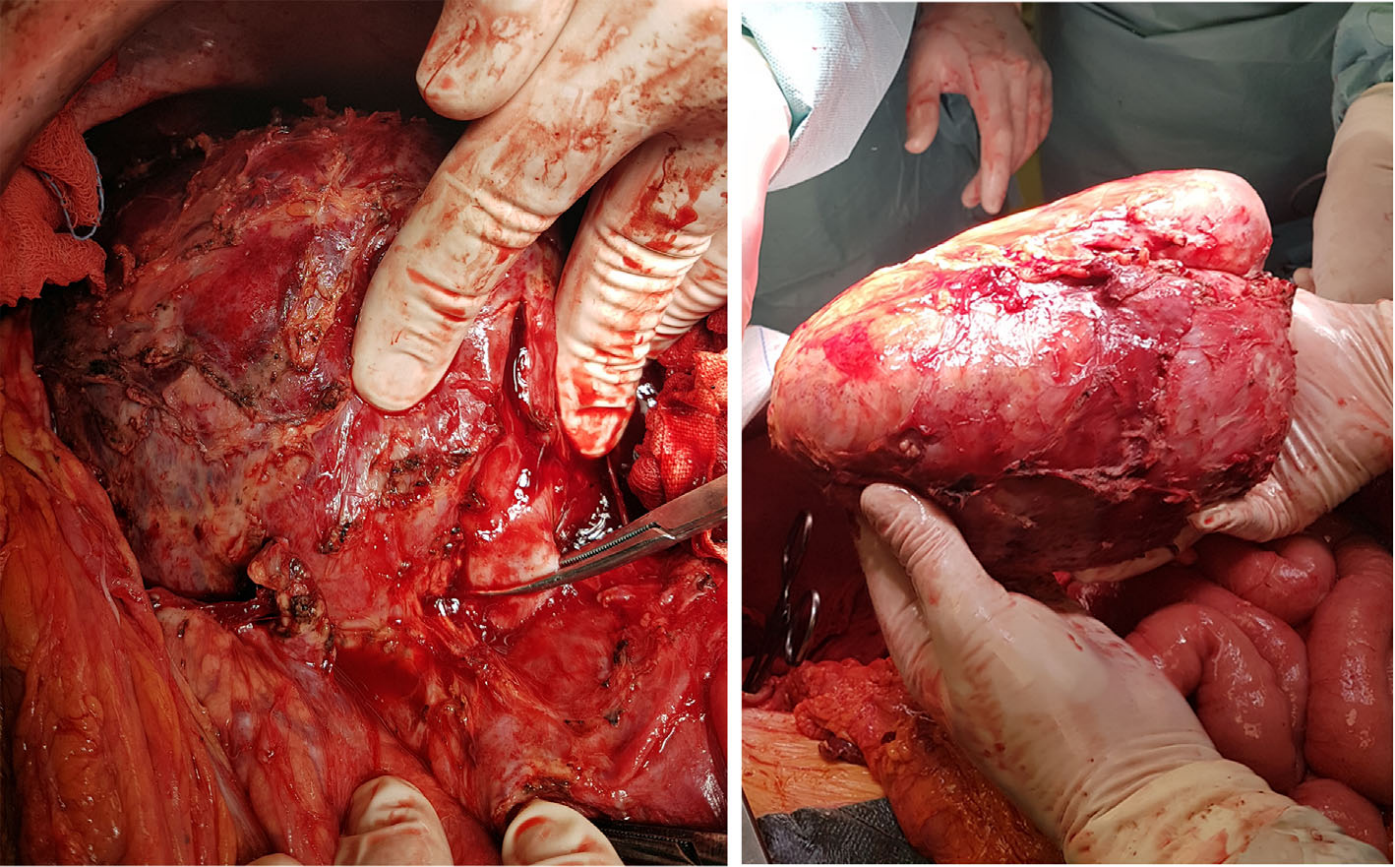
Surgical removal of the necrotic primary tumor together with the left kidney after resolution of the descending abscess. The left renal vein is clamped.

The CT documented a large necrotic mass occupying the tumor region (**Figure 1B**). Informed consent was obtained, and the patient was scheduled for surgical removal of the necrotic tumor after a planned 6-week-long, partly inpatient prehabilitation treatment to further reduce PEM. Before the operation in April 2017, however, a septic condition developed with a large intra-peritumoral abscess descending down to the left thigh through the femoral canal (**Figure 1C**). A left iliac venous thrombosis was also documented. Being too risky to be operated acutely, ultrasound-guided drainage of both the femoral (**Figure 1D**) and retroperitoneal regions (**Figure 1E**) was performed, prehabilitation continued, and therapeutic LMWH was again administered. The patient was stabilized and with repeated irrigations through the drains, the femoral abscess disappeared (**Figure 1D**), and the thrombosis resolved. Together with the resolution of the septic condition, oral and parenteral calorization, the patient gradually gained weight (4 kg). After 6 weeks, surgical re-exploration was performed in June 2017, and the large retroperitoneal mass was successfully resected together with the left kidney (R0) (**Figure 4**). The postoperative course was uneventful. Pathological result revealed gross tumor necrosis; only minor patches of non-tumorous suprarenal tissue islands could be identified. Viable tumor was not found.

The patient has been regularly followed-up since June 2017 (CT or MRI every 3-6 months) without proof of viable residuum or tumor recurrence. The small S8 scar in the liver is not growing and considered non-viable. There is no sign of novel metastasis. The laboratory parameters including kidney function, liver enzymes and blood count are in the normal range. The patient gained weight (BMI 20) and feels completely healthy. Adjuvant therapy has not been initiated any more. At present, the patient can be regarded as tumor free.

## Discussion and Conclusion

The biological nature of adrenocortical cancers is heterogeneous. Known risk factors are age, Ki-67-index, early presence of metastases, number of affected organs and R0 resection (1, 7). There is remarkable heterogeneity of individual tumors in the same stage that is well reflected by the variable survival in the groups. In a minority of cases, even long-term survival (>60 months) is possible with distant metastases, although the median survival is only 6-20 months in stage IV (4, 5, 8). Even large volume oligo-metastases, if resected, have much better prognosis than multiple small lesions, as the latter are usually poor candidates for any surgical or ablative therapy (6, 8).

The authors are unsure, whether the primary tumor would have been really unresectable, if the first attempt was done in a more experienced center; however, this first unsuccessful surgical exploration was a key factor in this series of partly unpredictable events showing that selective embolization of a primary ACC may lead to complete tumor necrosis. It is also very probable, that the patient would have later undergone liver resection, had she been referred to the university multidisciplinary team at the beginning. Going here through a very different path, a theoretically palliative method was proved to be curative.

Transarterial embolization of liver tumors, mainly hepatocellular cancers (10), colorectal metastases (11), neuroendocrine tumors (12), and sometimes others (13) have been routinely practiced worldwide in the last decades. However, there is limited experience available regarding adrenal embolization, especially for ACC. Adrenal embolization may carry some risk of catecholamine release, but usually only in phaeochromocytoma (14). Most adrenal embolizations in the literature are reports of sporadic cases of acute tumoral (mainly phaeochromocytoma, myelolipoma, metastases) or traumatic bleedings (15), palliative embolizations to reduce hormone secretion or for pain control (14), preoperative interventions to reduce vascularity before surgery (16), or more rarely palliative interventions to control tumor growth (14). Spontaneous bleeding associated with solid adrenal masses is rare. The number of reported cases in the literature was 139 till 2012 (17), and there have been only sporadic cases reported since then (18, 19). Primary adrenocortical cancer represents a minority among these, with approximately 7% of all events (17). The number of reported bleeding tumors largely exceeds the number of documented embolizations (15, 17): most probably only a minority of all patients are treated minimally-invasively.

The number of reported primary or locally recurrent ACCs treated with embolization is 6 in the English literature. O’Keeffe et al. (14) reported 4 embolizations for unresectable ACCs – biochemical response was reported, tumor burden was not documented, significant survival benefit was not achieved. Li et al. (19) reported a case of a locally recurrent and metastatic (liver, spleen) ACC, where three sequential embolizations resulted in partial response and 58 months survival of the patient. Somewhat similar to our case, a ruptured and bleeding adrenocortical cancer was treated by Kashiwagi et al. (20): embolization of the medial suprarenal artery was followed by surgical removal of the primary tumor after 2 months, and a wide necrotic region was documented in the partially affected tumor.

Treatment of ACC liver metastases should anecdotally have better results, but such cases are rarely reported. Koh et al. (21) documented partial remission after embolization of a solitary liver lesion but no survival benefit. Owen et al. (22) suggested from data of 6 patients that TACE or SIRT for liver metastases may contribute to better survival of their patients. Cazejust (23) et al. achieved stabilization or radiological regression in the majority of their patients in a larger series of 26 cases, but complete response was not achieved. However, there are few reports on complete tumor regression: Soga et al. (24) published two cases with TAE for solitary metastases, Wong et al. (25) successfully treated a patient with multiple hepatic tumors with TACE (trans-arterial chemoembolization), and recently SIRT (selective internal radiotherapy) was also reported to achieve complete tumor clearance in a patient with hepatic ACC metastasis (26).

The variable arterial supply of the adrenal gland may limit the effect of its embolization: each adrenal gland may be supplied from 3 major arteries arising from the aorta/celiac trunk, renal artery, subphrenic artery to form a strong plexus under the capsule (27), but most can be found with catheters. Most probably, there is no difference among the different types of embolizing agents for permanent adrenal interventions (15). In our case, one single dominant artery was documented and effectively treated, however, malignant tumors may develop collaterals through tumoral neoangiogenesis that may be impossible to reach (15). In our case, severe intratumoral hemorrhage itself may have caused necrosis or altered the blood flow; however, we think it played only a minor role, since the dominant artery was mapped and found before the embolization during the first, hepatic intervention and remained practically unchanged during the acute procedure. Beyond its anatomical properties, the biological nature of each individual tumor, their number, and capacity for embolizing agent uptake may also determine their potential response to TAE (15, 23). The value of a single case report, as this one, is always limited by its unique nature; but the number of embolized ACCs is so low in the literature, that the potential of this modality, based on our findings, might be stronger than thought before in this rare tumor type.

The necrotic areas affected by TAE might become infected. These events are considered to be rare (0-1%) in terms of liver tumors, where there is much more documented experience available (28, 29). Necrotic areas or abscesses may be treated with drainage and antibiotics effectively in the liver (29, 30). Regarding the adrenal gland, the organ is routinely removed after some time in most cases following embolization – most of these cases include acute bleedings (15) or preoperative devascularizing procedures (16). However, adrenalectomy will not necessarily be performed with palliative or traumatic background (14, 15). We were not aware of any report of adrenal abscess as a consequence of embolization in the literature. Patients generally do not experience more than low-grade fever and short-term mild flank pain after interventions (15). Although the large necrotic area together with the intratumoral hematoma in our case obviously carried risk of superinfection, the second surgical attempt was delayed because of the poor general condition of the patient and her preference.

About two-thirds of patients develop local recurrence or distant metastases, and most of these tumors are diagnosed in the first two postoperative years (3–6). The median recurrence-free interval is around 11 months (6). Follow-up criteria are not rigorously determined in the current ENSAT guideline or elsewhere (1); we chose the three months interval for CT or MRI (both recommended by ENSAT for the diagnosis for ACC) follow-up to surely not to miss a recurrence in this exceptional case.

In conclusion, we presented that not just liver metastases, but even a large primary ACC may be effectively treated by arterial embolization facilitating R0 resection. Based on the many times proved fact, that R0 resection is the key to survival in ACC, this example suggests a potentially more important role for preoperative embolization in ACC not just as a technical adjunct, but also as a potential method to improve oncological efficacy: transarterial embolization may represent an effective treatment modality in adrenocortical cancer.

## Data Availability Statement

The original contributions presented in the study are included in the article/supplementary material. Further inquiries can be directed to the corresponding author.

## Ethics Statement

Written informed consent was obtained from the individual(s) for the publication of any potentially identifiable images or data included in this article.

## Author Contributions

GH: operating surgeon, surgeon of endocrine-oncology multidisciplinary team, concept of work and manuscript. AD: interventional radiologist. KF: ward surgeon and critical review. LK: head of surgical department and critical review. PR: ward endocrinologist and critical review. JT: multidisciplinary team endocrinologist and critical review. MT: endocrine-oncologist, head of multidisciplinary team, and critical discussion of case. PI: endocrinologist, outpatient care and follow-up, revision of manuscript, and treatment supervision. All authors contributed to the article and approved the submitted version.

## Funding

Hungarian National Research, Development and Innovation Office (NKFIH) grant K134215 to PI. The study was also financed by the Higher Education Institutional Excellence Program to Semmelweis University by the Ministry of Human Capacities in Hungary.

## Conflict of Interest

The authors declare that the research was conducted in the absence of any commercial or financial relationships that could be construed as a potential conflict of interest.
